# Differential Branchial Response of Low Salinity Challenge Induced Prolactin in Active and Passive Coping Style Olive Flounder

**DOI:** 10.3389/fphys.2022.913233

**Published:** 2022-06-29

**Authors:** Junjia Zeng, Jie Li, Kun Yang, Jiayu Yan, Tianchun Xu, Weiqun Lu

**Affiliations:** ^1^ National Demonstration Center for Experimental Fisheries Science Education, Shanghai Ocean University, Shanghai, China; ^2^ The Key Laboratory of Exploration and Utilization of Aquatic Genetic Resources, Ministry of Education, Shanghai, China; ^3^ Southern Marine Science and Engineering Guangdong Laboratory, Guangzhou, China; ^4^ International Research Center for Marine Biosciences at Shanghai Ocean University, Ministry of Science and Technology, Shanghai, China

**Keywords:** salinity challenge, olive flounder, coping strategy, prolactin, ionocyte

## Abstract

Stress coping styles are very common in fish, and investigations into this area can greatly improve fish welfare and promote the sustainable development of aquaculture. Although most studies have focused on the behavioral and physiological differences of these fishes, the endocrine response of different coping styles fish when undergoing salinity challenge is still unclear. We examined the physiological response in olive flounder with active coping (AC) style and passive coping (PC) style after transferred from seawater (SW) to freshwater for 0, 2, 5, 8, and 14 days. The results showed that: 1) the plasma prolactin level of FW-acclimated AC flounder was substantially higher than that of FW-acclimated PC flounder at 5, 8, and 14 days, and the branchial gene expression of prolactin receptor (PRLR) in AC flounder was slightly higher than PC flounder after transfer. While there was no remarkable difference observed in cortisol (COR) levels between AC and PC flounder. After transfer, glucocorticoid receptor (GR) expression in AC flounder was significantly higher compared with PC flounder at 8 days. 2) Branchial NKA-IR ionocytes numbers were reduced in PC flounder after transfer, while ionocytes number remain stable in AC flounder. 3) The branchial stem cell transcription factor *foxi1* gene expression of AC flounder was significantly higher than PC flounder at 2, 5, and 14 days after transfer, while branchial stem cell transcription factor *p63* gene expression of FW-acclimated AC flounder was only substantially higher than that of PC flounder at 5 days. 4) As an apoptosis upstream initiator, the branchial gene expression of *caspase-9* in PC flounder was considerably higher than in AC flounder after transfer at 8 days. This study revealed that olive flounder with active and passive coping styles have different endocrine coping strategies after facing the low-salinity challenge. AC flounder adopt an active endocrine strategy by increasing ionocyte differentiation and prolactin secretion significantly. In contrast, PC flounder employ a passive strategy of reducing ionocytes differentiation and retaining prolactin content at a low level to reduce branchial ionocytes number.

## Introduction

Coping style can be defined as a coherent set of behavioral and physiological stress responses that are consistent over time and are characteristic of a certain group of individuals (Koolhaas et al., 1999). Active coping (AC) individuals and passive coping (PC) individuals are observed in olive flounder (*Paralichthys olivaceus*) (Rupia et al., 2016; Zeng et al., 2019). Compared to PC flounder, AC flounder show differences in response to simulated capture, feed propensity and metabolic rate (Rupia et al., 2016). For example, during acute stress, passive coping individuals adopt a passive “freeze-hide” strategy by reducing their oxygen consumption rates and remaining immobile, whereas active coping individuals adopt an active “fight-flight” defence strategy by increasing their rates of respiration and activity levels (Rupia et al., 2016). The majority of studies focused on the behavioral characteristics of different coping style individuals (Overli et al., 2006; Silva et al., 2010; Martins et al., 2011), and there are also some research investigating the physiological characteristics of individuals with different coping styles, such as hypothalamus-pituitary-interrenal (HPI) activity (Castanheira et al., 2013), immunity (Kittilsen et al., 2012) and hormonal modulation (LeBlanc et al., 2012). However, only a few studies elucidate the osmoregulatory coping differences between these two coping styles individuals (Sloman et al., 2003; Sloman et al., 2004), especially under salinity challenge (Zeng et al., 2019).

Salinity challenge is a common situation for fishes: with the increasingly climate change, resultant increase of extreme events, like hurricanes, heavy rainfall, and flooding, considerably fluctuated the water salinity (Bailey and Secor, 2016; Williams et al., 2017). Olive flounder, as a coastal habitat euryhaline fish, bears the brunt of salinity challenge (Yuan et al., 2017). Flounders also have a sophisticated osmoregulatory system in which the gills, the most important organ, take on the 90% function of osmoregulation that is mainly achieved by the ionoregulatory cells i.e., ionocytes (Evans et al., 2005). Ionocyes, formerly called chloride cells or mitochondrion-rich cells, are located at the base of the gill filament and among lamella. Fishes respond to salinity challenges through the function of ionocytes.

Body fluid homeostasis is vital for the survival of organisms and includes cellular activities and many physiological processes. Fish have developed a sophisticated endocrine system, mainly through hormones, to regulate body fluid homeostasis to meet environmental stressors such as salinity challenge. Hormones regulate body fluid homeostasis by controlling epithelial transporters. Several hormones have been demonstrated to positively or negatively regulate ion transport through specific receptors at transcriptional, translational or posttranslational levels, and at different stages of ionocyte development (i.e., proliferation or differentiation) (Hwang and Chou, 2013; Guh et al., 2015; Lin et al., 2016; Guh and Hwang, 2017; Lewis and Kwong, 2018). Cortisol plays many roles in physiological processes and is involved in regulating hydromineral balance in FW and SW teleosts (Guh et al., 2015). Cortisol also maintains ion transporters and ionocytes to promote both hyper- and hypo-osmoregulation mechanisms in the gill of fish (Evans et al., 2005). In teleosts, zebrafish are model organisms for studying regulatory pathways, which is of great significance to the study of osmotic pressure, and cortisol regulates Na^+^ uptake through glucocorticoid receptors (*GR*) in zebrafish (Kumai et al., 2012). Indeed, accumulated evidence has demonstrated a predominant role for cortisol-*GR* signaling in fish iono/osmoregulation (Takahashi and Sakamoto, 2013). Prolactin has long been known as an FW adapting hormone, it inhibits the formation of SW ionocytes and promotes the development of FW ionoocytes (Evans et al., 2005). Furthermore, prolactin has been linked to the regulation of Na^+^-Cl^−^cotransporter (NCC) expression and ionocyte differentiation (Guh et al., 2015). Prolactin receptors (*PRLR*) are also crucial in the osmoregulation of fish (Guh and Hwang, 2017), the expression level of *PRLR* in teleost gill is very high (Prunet et al., 2000; Tse et al., 2000; Santos et al., 2001).

*Foxi1*, as a forkhead transcription factor, plays a vital role in the mechanism of α-intercalated cells (IC) differentiation in the mammalian inner ear and kidney (Blomqvist et al., 2004). Previous research in teleost fish, however, revealed that *Foxi1* indirectly regulates the differentiation of “HR-type” ionocytes (Esaki et al., 2009). The transcription factor *p63* is a marker of stem cells in the mammalian epidermis (Horng et al., 2009). In zebrafish (*Danio rerio*), an isoform of *p63* promotes the proliferation of epidermal cells, and then epithelial stem cells differentiate into skin ionocytes (Horng et al., 2009). Salinity challenge can induce apoptosis of fish branchial epithelial cells (Ching et al., 2013). *Caspase-9* is an initiator caspase involved in the induced apoptosis of macrophages and neutrophils, an activity involving the intrinsic pathway (Awoyemi et al., 2019).

With the rapid development of the world’s aquaculture industry, total fish production has almost doubled during the last 20 years in Asia. Since the mid-2000s, Asia has accounted for two-thirds of global inland production (data from “The State of World Fisheries and Aquaculture 2020″). Indeed, annual flounder production has been roughly estimated to be 28,000 metric tons since the 2000s in Japan, Korea, and China, and therefore, flounder culture has become an important economic source of aquaculture in these areas (Seikai, 2002). Studies on the coping styles of farmed fish can provide knowledge for sustainable aquaculture, provide advantages in the culture system, and could be utilized to establish selection-based breeding programs to improve domestication and production (Ibarra-Zatarain et al., 2016; Fatsini et al., 2020).

Previous studies have shown that olive flounder with AC and PC styles have different coping strategies of behavioral and physiological response during FW acclimation (Zeng et al., 2019). However, the response of endocrine system of olive flounder with different coping styles to salinity challenge are still unclear. To fill this gap, therefore, our study measured the following parameters of olive flounder with AC and PC styles after transferred from SW to FW: 1) the plasma cortisol and prolactin concentrations and gene expression of their corresponding receptors (i.e., *GR* and *PRLR*) in fish gills, 2) the density of branchial ionocytes, and 3) the gene expression of branchial *foxi1*, *p63* and *caspase-9*.

## Materials and Methods

### Experimental Fish

The gynogenetic olive flounder were cultured separately in SW recirculating aquaculture systems at the Central Experimental Station of Chinese Academy of Fisheries Sciences in November 2018 (Beidaihe, Hebei, China). According to the previous method, the fish (body weights: 500 ± 50 g) were screened through behavior tests which including air exposure and feeding propensity and then divided into AC groups and PC groups, the specific experimental method has been elaborated in the previous article (Rupia et al., 2016; Zeng et al., 2019). Olive flounder with confirmed active coping style (AC, *n* = 48) and confirmed passive coping style (PC, *n* = 48) were distributed evenly into 16 holding tanks (water capacity = 500 L; *n* = 6 for each coping style in each tank) and maintained for 2 weeks with filtered flow-through SW at a salinity level of 30‰. Water temperature was maintained at 18 ± 1°C, with a constant 12 h light/12 h dark cycle (8:00 a.m.—20:00 p.m.). The mean light intensity, measured centrally at the bottom of each tank, was approximately 40 lux (Zeng et al., 2019). Before the experiment, the fish were fed to satiation twice daily (8:00 a.m. and 18:00 p.m.) with commercial fish pellets (“Wenger” High-Grade Aquatic Feed 6 mm; Wengzhuang, Beijing, China). After that, the fish were fasted throughout the experiment to ensure that differential rates of feeding did not influence the results of the study (Yuan et al., 2017).

### The Gill Characteristics of AC and PC Coping Styles to Salinity Challenge

Olive flounder with active coping style (AC, *n* = 48) and passive coping style (PC, *n* = 48) were netted and divided into four seawater (SW, 30‰, control group) tanks and four freshwater (FW, 30‰) tanks on the basis of corresponding coping styles (*n* = 6 for each coping style in each tank). The SW-acclimated (*n* = 3 in each tank) and FW-transferred (*n* = 3 in each tank) fish were then sampled during daylight hours 0, 2, 5, 8, and 14 days after initial transfer. At each sampling point, fish were rapidly netted and anesthetized with 2-phenoxyethanol (0.2 ml/L, Sigma, St. Louis, MO), and blood samples (3–5 ml) were collected within 90 s using a heparinized needle and syringe (200 U/ml heparin, Sigma) by caudal venipuncture. Blood was then aliquoted into ammonium-heparinized tubes (200 U/ml, Sigma) and plasma was separated by centrifugation for 5 min at 13,000 rpm and stored at −80°C for the subsequent measurement of plasma hormone concentrations. The fish were then sacrificed humanely by severing the spinal cord and destruction of the brain. A part of the gill was removed from the first gill arch and stored at −80°C for later analysis of branchial gene expression. A part of the gill was also removed and fixed in 4% paraformaldehyde (PFA) for 24 h for immunohistochemistry.

### Plasma Measurements

Plasma levels of cortisol (COR) and prolactin (PRL) were quantified by ELISA commercially (Qiyi Biotechnology Co., LTD., Shanghai, China). According to the manufacturer’s instructions, the circulating level range of COR was detected between 4 and 15 ng/ml, PRL was detected between 500 and 3,000 ng/ml. In detail, −80°C stored plasma supernatant fractions were naturally warmed in an icebox. Samples were then diluted to the appropriate concentration. Commercially available ELISA kits (Qiyi biotechnology Co., LTD., Shanghai, China) were subsequently used to measure serum COR and PRL levels in duplicate as per manufacturer instructions (Mechesso, 2019).

### Antibody

Rabbit polyclonal anti-alpha 1 subunit of Na^+^/K^+^-ATPase (Abcam, Cambridge, United Kingdom) was used to detect the NKA immunoreactive (NKA-IR) ionocytes in the gill. The commercial polyclonal NKA-α1 antibody used in present study was specialized to zebrafish’s NKA-α1 (Hiroi and McCormick, 2012). We blast the protein and nucleotide sequence of the zebrafish’s NKA-α1 with that of olive flounder, and the results showed highly homologous of 92% and 84%, respectively. The secondary antibody for immunohistochemistry was goat anti-mouse IgG (Thermo Fisher Scientific, United States). Na^+^/K^+^-ATPase NKA, as the main Na^+^ transporter, has been localized to the basolateral region of ionocytes. These NKA antibodies serve as a highly-specific marker for ionocytes in fixed gill tissues (Hiroi and McCormick, 2012).

### Immunohistochemical Detection and Analysis of NKA Immunoreactive Cells

The Paraformaldehyde-fixed gills were dehydrated in ethanol, cleared in xylene and embedded in paraffin. A cross section of the fish gills was cut at 6 μm thickness on a microtome and mounted on glass slides, which were dealt with positive charge. During the experiment, tissue sections were dewaxed in xylene and rehydrated in gradient alcohol. Endogenous peroxidase activity was blocked with 3% H_2_O_2_ in methanol before slides were placed in 0.01 M citrate buffer and heated in a water bath for 20 min at 95°C. Sections were rinsed in PBS after cooling. Sections were treated with fetal bovine serum (FBS) blocking solution (1% blocking, dissolved in MABT, and 5% FBS in PBST, PBS with 0.1% Triton X-100) for 1 h at room temperature (RT) to reduce nonspecific staining and incubated with 1:500 rabbit polyclonal anti-alpha 1 subunit of Na^+^/K^+^-ATPase diluted with PBS in a moist chamber at 4°C overnight. Next day, the moist chamber was transferred into air oven at 37°C for 45 min, then washed six times at RT in PBST for 15 min each time and incubated with 1:500 goat anti-rabbit IgG secondary antibody. Then the sections were washed thrice in PBST. Control experiments were carried out by omission of the primary antibody and pre-absorption of the antibody with an excess of antigenic peptide.

### Quantification of Branchial Ionocytes

The number of NKA immunoreactive (NKA-IR) ionocytes were counted and obtained within each gill in every five sections (five adjacent lamellas were set as one section) from all sections sampled at ×200 magnification with a microscope (Nikon ECLIPSE 55i, Nikon Corporation, Japan). Size and luminosity of the figures were modified with NIS-Elements Version 4.0 (Nikon, Japan), as well as the drawings.

### The Branchial Gene Expression Response of AC and PC Coping Styles to Salinity Challenge

Extracted Total RNA from the samples of gills using RNAiso Plus (Takara, Japan) and then reverse transcribed the total RNA into cDNA using PrimeScript™ RT reagent (Takara, Dalian, China) according to the standard method. Relative quantification of the target gene transcripts was analyzed using *β-actin* gene expression as the reference gene (Zeng et al., 2019). Branchial *foxi1*, *p63*, *caspase-9*, *GR* and *PRLR* gene expressions were determined using ABI 7500 (Applied Biosystems, Carlsbad, CA) with SYBR Premix Ex Taq™ (Takara, Dalian, China) and the following PCR conditions were used: 10 min at 95°C, followed by 36 cycles of 95°C for 10 s, 30 s at 60°C. Gene sequences were obtained from NCBI and all the primers were designed by NCBI primer blast. The forward and reverse primers span an exon-exon junction. All the primers were tested and shown to be viable and specific. The primer sets for the quantitative RT-PCR are shown in Table 1.

**TABLE 1:** Primer sequences used for real-time PCR amplifications.

Genes	Primer (5′–3′)	Gene bank	Product length (bp)
*β-actin*	GGA​AAT​CGT​GCG​TGA​CAT​TAA​G	HQ386788.1	161
	CCT​CTG​GAC​AAC​GGA​ACC​TCT		
*GR*	ACC​AGT​TTA​ACG​CGG​TTT​GC	XM_020103452.1	261
	ACG​CCA​CAT​TTA​TCC​ATC​CTT​G		
*PRLR*	CCC​GAC​TAT​TTC​CAT​CGG​GAG	XM_020081654.1	247
	AGT​TGG​ACG​ATG​TGA​TCG​GG		
*Foxi1*	TGA​CCC​AGG​AAA​GGG​CAA​CTA	XM_020086875.1	262
	CAA​GAA​GCT​GCT​CAG​ACA​CG		
*P63*	CTC​CCC​AGG​TTG​GAA​CAG​AA	XM_020080690.1	253
	CGT​TTC​GTA​CCC​TCA​CTG​CT		
*caspase-9*	AAG​ACA​CTG​ACT​GAC​TCC​GC	XM_020089046.1	257
	GTC​TGG​TCC​CAG​AGC​TCT​TAA​T		

### Statistical Analysis

The 2^−ΔΔ Ct^ method was used to analyze the quantitative RT-PCR data, and the expression level in all plots of each gene is presented as the relative change in values from SW-acclimated AC at 14 days, which was set at 1 (Zeng et al., 2019). The normality and homogeneity of variance of data were tested using Shapiro-Wilk’s test and Levene’s test, respectively. Significant analyse of plasma hormone was assessed using nonparametric test with salinity (or coping style) and time as independent variables, followed by Mann-Whitney U test. Significant analyses of branchial inocytes numbers and gene expressions were assessed using two-way ANOVA with salinity (or coping style) and time as independent variables, followed by Tukey’s multiple comparison test. Data are expressed as means ± SEM throughout and significance was accepted at *p* < 0.05 for all analyses. All statistical analysis was carried out using the SPSS Statistics 20 or GraphPad Prism 5.0 (San Diego, CA, United States).

## Result

### Plasma Hormone Level and Its Branchial Receptor Gene Expression

**FIGURE 1:**
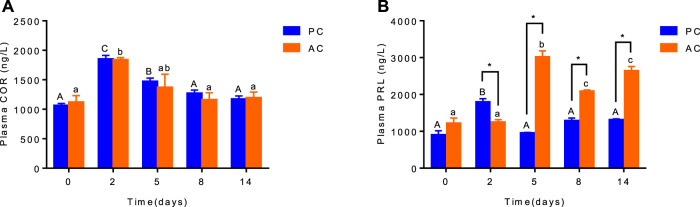
The plasma cortisol **(A)** and prolactin **(B)** level in olive flounder with passive coping style (PC) and active coping style (AC) after transfer to fresh water. Data are mean ± SEM (*n* = 3). An asterisk signifies a significant difference between the PC and AC groups at each time point (*p* < 0.05). Different letters denote a significant difference across time within the same coping style group (*p* < 0.05).

There was no interaction between coping style and time on plasma cortisol level (ANOVA, F_(4,20)_ = 0.1487, *p* = 0.9614, Figure 1A). While the plasma cortisol levels of PC and AC flounder increased from 0 to 2 days and decreased from 2 to 14 days during FW acclimation (*p* < 0.05; Figure 1A). Significant interactions between coping style and time were detected for plasma prolactin level (ANOVA, F (4,20) = 58.95, *p* < 0.0001, Figure 1B), indicating that prolactin response were different among AC and PC at different time points. Significant effects of treatment and time on AC flounder plasma PRL were observed, with a significant increase from 2 to 5 days (*p* < 0.05, Figure 1B), and significantly decreased thereafter (*p* < 0.05, Figure 1B). While PC flounder were significantly increased from 0 to 2 days (*p* < 0.05, Figure 1B), significantly decreased from 2 to 5 days (*p* < 0.05, Figure 1B), and then remained stable. After FW transfer, the PRL levels of AC flounder were significantly lower than PC flounder at 2 days (*p* < 0.05, Figure 1B), but significantly higher than PC flounder at 5, 8 and 14 days (*p* < 0.05, Figure 1B).

**FIGURE 2:**
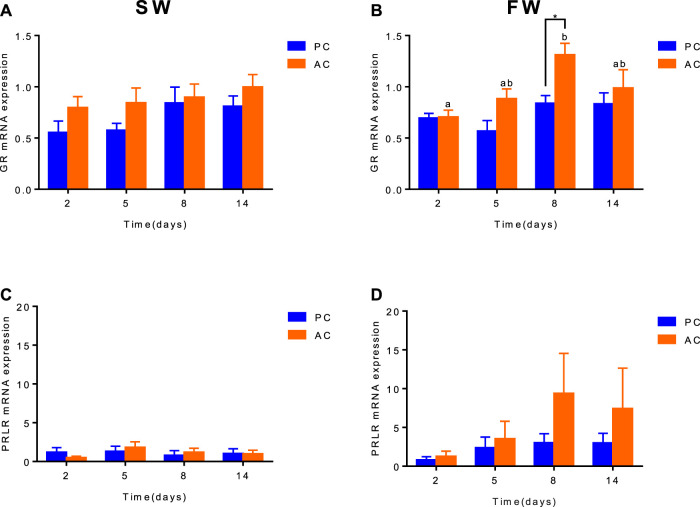
The branchial gene expression of *GR* **(A,B)** and *PRLR* **(C,D)** in olive flounder with passive coping style (PC) and active coping style (AC) either in seawater (SW, **A,C**), or after transfer to freshwater (FW, **C,D**). Data are mean ± SEM (*n* = 6). An asterisk signifies a significant difference between the PC and AC groups at each time point (*p* < 0.05). Different letters denote a significant difference across time within the same coping style group (*p* < 0.05).

When acclimated to SW, there was no difference in *GR* and *PRLR* gene expression between olive flounder with both active and passive coping style (Figures 2A,C). After transferring to FW, the *GR* expression of AC flounder significantly increased up to 8 days (2 days vs*.* 8 days, *p* = 0.0039), and slightly decreased at 14 days, and was significantly higher than that of PC flounder at 8 days (*p* = 0.0467, Figure 2B). While FW-acclimated PC flounder showed no remarked changes in *GR* gene expression (Figure 2B). The *PRLR* expression in FW-acclimated AC flounder tented to be slightly higher than that of PC flounder from 2 to 14 days, but without significant difference (Figure 2D).

### Branchial Ionocytes Number of AC and PC Flounder

**FIGURE 3:**
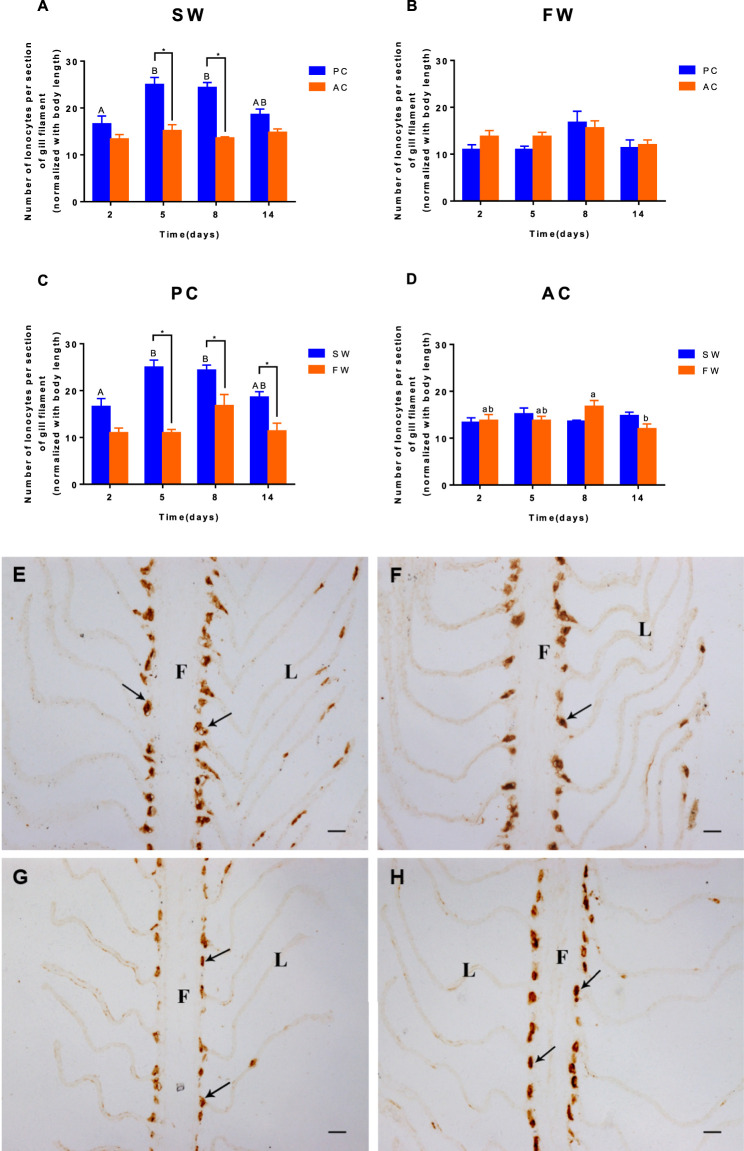
The number of ionocytes per section of gill from olive flounder with passive coping style [PC, **(C)**] and active coping style [AC, **(D)**] after transfer from seawater [SW, **(A)**] to freshwater [FW, **(B)**]. Five adjacent lamellas were set as one section. Each gill of a fish chose five sections. Data are mean ± SEM (*n* = 6). An asterisk signifies a significant difference between the PC and AC groups at each time point (*p* < 0.05). Different letters denote a significant difference across time within each PC or AC group (*p* < 0.05). The immunocytochemistry for ionocytes in gills from olive flounder with passive coping style [PC, **(E,G)**] and active coping style [AC, **(F,H)**] from seawater [SW, **(E,F)**] to freshwater [FW, **(G,H)**] for 5 days. Scope time (×200). F, filament; L, lamella; black arrow, and ionocytes. Bar = 20 μm.

Significant interactions between salinity and time were observed for the branchial ionocytes number of PC and AC (ANOVA, F_(3,32)_ = 7.934, *p* = 0.0004, Figure 3A; ANOVA, F (3,32) = 5.952, *p* = 0.0024, Figure 3B). When acclimated to SW, the branchial ionocytes numbers of PC siginificantly increased from 2 to 5 days (2 days vs. 5 days, *p* = 0.0003, Figure 3A) and then remained unchanged. No significant differences were found in FW-acclimated AC and PC (Figure 3B). Moreover, we observed that compared with the SW control group, the number of NKA-IR ionocytes in fish gills was counted and showed dramatic decrease in FW-acclimated PC individuals from 2 to 14 days (*p* < 0.05, Figure 3C). In addition, the cell number of AC individuals remained unchanged regardless of whether it was acclimated to SW or FW (Figure 3D). The immunocytochemistry for ionocytes in gills of AC (Figures 3F,H) and PC (Figures 3E,G) flounder transferred from SW (Figures 3E,F) to FW (Figures 3G,H) also showed this phenomenon.

### Gene Expression of Branchial *foxi1* and *p63*

**FIGURE 4:**
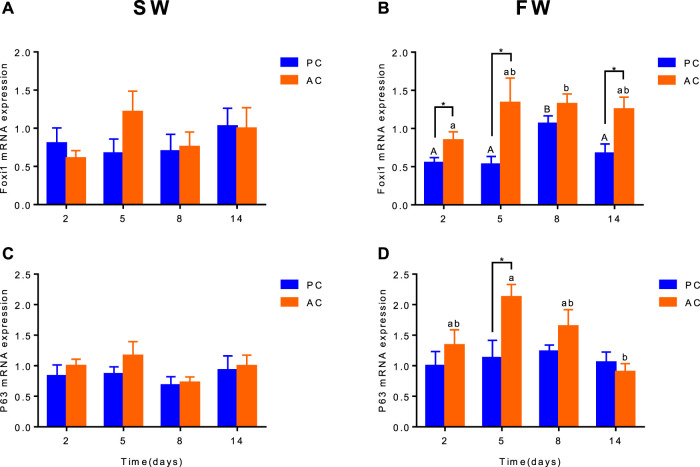
The branchial gene expression of *Foxi1* **(A,B)** and *P63* **(C,D)** in olive flounder with passive coping style (PC) and active coping style (AC) either in seawater [SW, **(A,C)**], or after transfer to freshwater [FW, **(C,D)**]. Data are mean ± SEM (*n* = 6). An asterisk signifies a significant difference between the PC and AC groups at each time point (*p* < 0.05). Different letters denote a significant difference across time within the same coping style group (*p* < 0.05).

Branchial gene expression of *foxi1* and *p63* of SW-acclimated PC flounder were similar to that of AC flounder, each group did not show significant differences between PC and AC flounder at different time points. Nevertheless, main effect of coping style was observed for *foxi1* (ANOVA, F_(1,40)_ = 19.57, *p* < 0.0001, Figure 4B) and *p63* (ANOVA, F (1,40) = 7.108, Figure 4D) gene expression in FW-acclimated groups. When acclimated to freshwater, *foxi1* gene expression of AC was significant increased at 2 days (*p* = 0.038, Figure 4B), 5 days (*p* = 0.0368, Figure 4B) and 14 days (*p* = 0.01443, Figure 4B) compared with PC, both PC and AC flounder had peak expression of *foxi1* at 8 days (2 days vs. 8 days: PC, *p* = 0.0015; AC, *p* = 0.0173; Figure 4B). While *p63* gene expression of FW-acclimated PC was only significantly different from that of AC at 5 days (*p* = 0.035, Figure 4D). FW-acclimated AC also reach the peak expression of *p63* at 5 days, and then dropped significantly at 14 days (5 days vs. 14 days, *p* = 0.0044, Figure 4D).

### Gene Expression of Branchial Apoptosis Factor *Caspase-9*

**FIGURE 5:**
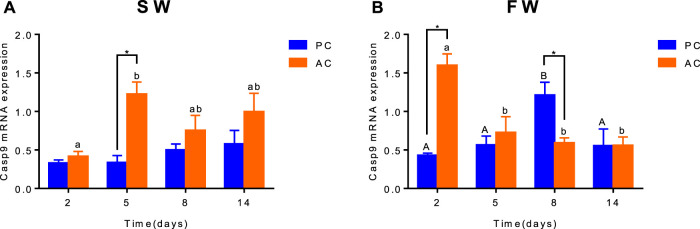
The branchial gene expression of *Caspase-9* in olive flounder with passive coping style (PC) and active coping style (AC) after transfer from seawater to seawater [SW, **(A)**] and freshwater [FW, **(B)**]. Data are mean ± SEM (*n* = 6). An asterisk signifies a significant difference between the PC and AC groups at each time point (*p* < 0.05). Different letters denote a significant difference across time within the same coping style group (*p* < 0.05).

Significant interactive effect between coping style and time were observed for *caspase-9* expression in SW (ANOVA, F_(3,40)_ = 2.882, *p* = 0.0476, Figure 5A) and FW (ANOVA, F_(3,40)_ = 13.35, *p* < 0.0001, Figure 5B), indicating that gene expression of *caspase-9* in flounder was different among coping style at different time points. There was a significant difference of *caspase-9* expression between SW-acclimated AC and PC at 5 days (*p* = 0.002, Figure 5A). And this gene expression of SW-acclimated AC flounder increased from 2 to 5 days (*p* = 0.0063, Figure 5A). After transferred to FW, significant differences of *caspase-9* expression were also observed between AC and PC flounder at 2 days (*p* < 0.0001, Figure 5B) and 8 days (*p* = 0.005, Figure 5B), respectively. FW-acclimated PC flounder increased gene expression from 2 days, and reached peak expression at 8 days (2 days vs. 8 days, *p* = 0.009, Figure 5B), declined thereafter (8 days vs. 14 days, *p* = 0.0453, Figure 5B). While FW-acclimated AC flounder expressed highest gene expression at 2 days (2 days vs. 5 days, *p* = 0.0025, Figure 5B) and declined thereafter (Figure 5B).

## Discussion

Our study demonstrates marked differences in the hormone and ionocyte regulation strategies of PC and AC olive flounders in response to salinity challenge. PC flounder reduced branchial ionocytes density while AC flounder remain stable during hypoosmotic challenge. Also, FW-acclimated AC flounder have higher ultimate plasma prolactin levels, slightly higher branchial gene expression of hormone receptors (i.e., *PRLR* and *GR*), and higher ionocyte differentiation-related genes (i.e., *foxi1* and *p63*) than PC flounder. This is therefore the first study showing that ionocyte coping strategies influence the way fish deal with salinity challenge.

Many works had shown that cortisol not only function at SW-adaption but also important in FW-osmoregulation (Pelis and McCormick, 2001; Evans, 2002). Previous studies have also shown that cortisol treatment increases the densities of progenitor and mature ionocytes in larvae or isolated adult gills (Cruz et al., 2013a; Cruz et al., 2013b; Guh and Hwang, 2017). The pathway through which cortisol acts via the glucocorticoid receptor (*GR*) has been demonstrated to play a major role in fish osmoregulation (Takahashi and Sakamoto, 2013) and mediates the development of epidermal ionocytes in zebrafish (Cruz et al., 2013b). In the present study, the plasma cortisol level of both PC and AC increased from 0 to 2 days and gradually decreased from 2 to 14 days after transfer to FW, similar to previous studies of this species (Bolasina et al., 2007). Such results may be due to the rapid osmoregulatory action of cortisol in the low salinity challenge (Cruz et al., 2013a). However, after transfer to FW, AC flounder significantly increased *GR* expression, whereas no changes were found in PC. Study in zebrafish demonstrated cortisol-*GR* favors differentiation of ionocyte progenitors, thereby facilitating proliferation of mature ionocytes (Cruz et al., 2013a). Considering that the *GR* expression of AC flounder is higher than that of PC flounder after transfer to FW, there is such a possibility that AC flounder may have a higher rate of ionocytes development than PC during FW acclimation.

Prolactin, as a major “FW-adaption” hormone, promotes the development of “FW-type” ionocytes (Evans et al., 2005). Previous studies have shown that the plasma prolactin levels of FW-acclimated olive flounder were increased and remained stable from 2 to 14 days (Higashimoto et al., 2001; Yuan et al., 2017). Similarly, in our study, AC flounder also increased plasma prolactin concentration after transfer to FW from 2 to 5 days and restored stability at 14 days. However, PC flounder only showed an initial increase at 2 days and then kept to a lower level. Such results indicated a difference in hormone regulation between two coping style populations. Previous studies showed that FW treatment could increase branchial *PRLR* expression in olive flounder (Higashimoto et al., 2001; Yuan et al., 2017). Similar results were found in the present study, suggesting the role of *PRLR* in the osmoregulation process. Interestingly, during FW adaptation, both populations showed similar trends of the *PRLR* expression during acclimation to FW. However, the AC flounder *PRLR* expression was slightly higher than the PC flounder. Previous studies showed that AC individuals adopt an “active resistance” defense strategy to retain the plasma composition during hypoosmotic challenge, which was underpinned by a gradually increasing level of osmoregulatory gene expression in the gills for hypoosmotic adaptation; PC individuals employed a “passive tolerance” strategy to decrease the plasma osmolality and ionic content, which was underpinned by a sharp increase in the expression of branchial osmoregulatory gene, to conserve energy with the osmoregulatory process (Zeng et al., 2019). Moreover, the hormone synthesis is an energetically demanding bioprocess (Tseng and Hwang, 2008), Since the AC had higher metabolic space than PC flounder (Rupia et al., 2016; Zeng et al., 2019), the AC group possibly produce more hormone, which might explain the difference of prolactin level.

During adaptation to FW, NKA-immunoreactive (NKA-IR) ionocytes are mainly located on the filaments of AC and PC flounder. In this study, PC flounder reduced gill ionocytes density after transfer to FW, while that of the AC flounder remained unchanged, which means different ionocytes coping strategies between the two populations. Previous studies have found that, most euryhaline teleost fish, including chum salmon (*Oncorhynchus keta*) and Mozambique tilapia (*Oreochromis mossambicus*), as PC flounder in this study, showed significantly higher numbers of branchial NKA-IR ionocytes in SW than FW (Katoh and Kaneko, 2003; Hiroi and McCormick, 2012; Maugars et al., 2018). And tiger puffer (*Takifugu rubripes*), a stenohaline marine fish that has no change in the gill ionocytes activity after being transferred from a hypertonic to a hypotonic environment (Motohashi et al., 2009) which can also be seen in AC flounder. Similarly, our previous study found that the pattern of plasma parameters in FW-acclimated PC flounder showed a significant decrease and is similar to that seen in other euryhaline teleosts. In contrast, the pattern of plasma parameters in FW-acclimated AC flounder showed a fluctuation that is similar to the response seen in stenohaline marine fish (Zeng et al., 2019). Consequently, such results further confirm our former conclusion that markedly different hypoosmotic-regulation processes (i.e., ionocytes and osmoregulatory coping strategies) exist between AC and PC flounders. In addition, on one hand, a previous study found that AC flounder have a higher metabolic space than PC flounder (Rupia et al., 2016), meaning that AC flounder are accessible to spending more metabolic costs on osmoregulation, which is a much more energetically demanding process (Zeng et al., 2019). Therefore, AC individuals could cater to the salinity challenge to remain branchial ionocytes while PC flounders have to reduce cell numbers due to lower metabolic space. On the other hand, previous studies have also shown that PRL-deficient zebrafish down-regulate the density of “NCC-type” ionocytes (i.e., a type of NKA-IR ionocytes) and are unable to survive in FW (Shu et al., 2016). Thus, higher prolactin levels in AC flounder are beneficial for maintaining branchial “NCC-type” ionocytes density, and lower prolactin levels will assist in reducing the density in PC flounder. Prolactin plays its role by binding to the extracellular domain of the high-affinity receptors (i.e., *PRLR*), which are anchored on the membrane of the target cells (Bole-Feysot et al., 1998). Hence, due to the higher prolactin level and *PRLR* expression in AC, AC flounder may have a higher ionocytes development rate than PC flounder when responding low salinity challenge, maintaining ionocytes density as a consequence. Notably, when flounder maintained in SW, we observed that the branchial ionocytes number in PC was higher than AC. Previous studies indicated that the plasma cortisol level of SW-adaption PC flounder was higher than that of AC flounder (Zou et al., 2019). And cortisol was originally known as a SW-adaption hormone and promoted the differentiation of SW-type ionocyte (Evans et al., 2005). Therefore, such phenomena might be related to the higher plasma cortisol levels in PC than AC in SW.

*Foxi1*-deficient mouse mutants showed a defect in IC (α-intercalated cells) differentiation, demonstrating an important role for *foxi1* in establishing the IC lineage (Chang and Hwang, 2011). In zebrafish, *foxi1* could indirectly regulate the differentiation of epidermal stem cells into ionocytes (Esaki et al., 2009; Horng et al., 2009; Guh and Hwang, 2017). In the present study, after transfer to FW, *foxi1* gene expression of AC flounder was significantly higher than PC flounder at 2, 5, and 14 days, which may have resulted in a different cell differentiation process between AC and PC flounder. Ionocytes progenitors are also specified from *p63* positive epidermal stem cells and blocking ∆N*p63* (an isoform of *p63*) function in zebrafish embryos stopped the proliferation of epidermal cells (Lee and Kimelman, 2002). In this study, the gene expression of *p63* in FW-acclimated AC flounder showed a continuous increase and was significantly different compared with FW-acclimated PC flounder, indicated that AC flounder may induce epidermal stem cells at a higher proliferative rate than PC flounder when in response to low salinity challenge. So far, such a model is clearly constructed in zebrafish embryos: epidermal stem cells that express *p63* and *foxi1* give rise to ionocyte progenitors, which subsequently differentiate into ionocytes (Breves et al., 2014; Guh and Hwang, 2017). Previous studies suggest that prolactin may act on ionocyte progenitors to drive differentiation of ionoctes (Breves et al., 2014). Thus, we propose such a hypothesis that as the higher expression of ionocyte differentiation-related genes (i.e., *p63* and *foxi1*) in AC flounder stimulates the proliferation of epidermal stem cells, they give rise to ionocyte progenitors, accompanied by the higher prolactin level in AC flounder. Therefore promoting the differentiation of ionocyte progenitors into ionocytes, maintaining branchial ionocytes density.

Salinity stress may induce the proapoptotic caspase system (including *Casp-9* up-stream and *Casp-3* downstream) which executes apoptosis in oliver flounder (Lee et al., 2022). The apoptotic pathways are mediated by caspases, and as such, cells dying without the participation of caspases do not display the typical morphological characteristics of apoptosis. *Capsase-9* is an initiator of caspase family of cysteine proteases that have been implicated in apoptosis and cytokine processing (Takle and Andersen, 2007). Previous studies showed *caspase-9* is apparently involved in the induced apoptosis of macrophages and neutrophils, an activity involving the intrinsic pathway (Reis et al., 2007; Awoyemi et al., 2019). Salinity challenge could induce branchial cell apoptosis in fish (Li et al., 2019), as did low-temperature stress (Chou et al., 2008). In this study, FW acclimation led to a significant increase in branchial *caspase-9* gene expression in PC flounder, suggesting that the intrinsic pathways of apoptosis have been activated (Ching et al., 2013), and that the apoptosis rate of AC flounder may be different from that of PC flounder. The notable difference between AC and PC in 5 days after transfer to SW may be explained by the different coping style strategies between two groups. AC individuals always performed active response than PC in SW, therefore could initiate many complicated cellular mechanisms. The reason for this may be due to more intense energy consumption and physiological metabolism in AC individuals. The lower apoptosis rate in AC flounder may explain the distinct ionocytes density compared with PC flounder, which supports our hypothesis described above.

## Conclusion

Olive flounder with active coping style or passive coping style have markedly different endocrine coping strategies. When acclimated to FW, PC flounder reduced branchial ionocyte density while AC flounder remained stable. Also, compared with FW-acclimated PC flounder, that of AC flounder have higher branchial gene expression of ionocyte differentiation-related genes (i.e., *foxi1* and *p63*) and hormone receptors (i.e., *GR* and *PRLR*), higher plasma prolactin levels, and lower apoptosis upstream initiator *caspase-9* gene expression. As such, we suppose that AC flounder may have a higher epidermal stem cell proliferation rate, ionocyte progenitor differentiation rate, and ionocytes development rate than PC flounder when facing the low salinity challenge. These clearly show the different endocrine regulatory strategies between AC and PC.

## Perspective

The endocrine system is the major signaling pathway for environmental osmotic stress. When the animal is in an osmotically extreme environment, environmental osmotic stress may cause differences in endocrine response and control even among intraspecies (McCormick and Bradshaw, 2006). Our study has shown that olive flounder with different coping styles have different endocrine responses when acclimated to FW. However, a previous study showed that SW-type and FW-type ionocytes in fish gills could rapidly and reversely transform to accommodate salinity change (Hwang and Lee, 2007). Moreover, gills may sustain their functions by producing mature ionocytes from pre-existing undifferentiated progenitors in low-temperature environments (Chou et al., 2008). Therefore, further research is needed on the cell types and dynamic changes of ionocytes in flounder gill in response to a low salinity challenge.

## Data Availability

The original contributions presented in the study are included in the article/Supplementary Materials, further inquiries can be directed to the corresponding author.
